# Changes in Calcium Homeostasis and Gene Expression Implicated in Epilepsy in Hippocampi of Mice Overexpressing *ORAI1*

**DOI:** 10.3390/ijms20225539

**Published:** 2019-11-06

**Authors:** Lukasz Majewski, Bartosz Wojtas, Filip Maciąg, Jacek Kuznicki

**Affiliations:** 1 Laboratory of Neurodegeneration, International Institute of Molecular and Cell Biology in Warsaw, Trojdena 4, 02-109 Warsaw, Poland; fmaciag@iimcb.gov.pl (F.M.); jacek.kuznicki@iimcb.gov.pl (J.K.); 2 Laboratory of Molecular Neurobiology, Nencki Institute of Experimental Biology, PAS, Pasteura 3, 02-093 Warsaw, Poland; b.wojtas@nencki.gov.pl

**Keywords:** epileptic seizures, ORAI1, STIM1, STIM2, nSOCE, RNAseq, RT-PCR, Cdkl5, Arx, Dclk1

## Abstract

Previously, we showed that the overexpression of *ORAI1* calcium channel in neurons of murine brain led to spontaneous occurrence of seizure-like events in aged animals of transgenic line FVB/NJ-Tg(ORAI1)Ibd (Nencki Institute of Experimental Biology). We aimed to identify the mechanism that is responsible for this phenomenon. Using a modified Ca^2+^-addback assay in the CA1 region of acute hippocampal slices and FURA-2 acetomethyl ester (AM) Ca^2+^ indicator, we found that overexpression of *ORAI1* in neurons led to altered Ca^2+^ response. Next, by RNA sequencing (RNAseq) we identified a set of genes, whose expression was changed in our transgenic animals. These data were validated using customized real-time PCR assays and digital droplet PCR (ddPCR) ddPCR. Using real-time PCR, up-regulation of hairy and enhancer of split-5 (*Hes-5)* gene and down-regulation of aristaless related homeobox *(Arx)*, doublecortin-like kinase 1 (*Dclk1),* and cyclin-dependent kinase-like 5 (*Cdkl5*, also known as serine/threonine kinase 9 (*Stk9*)) genes were found. Digital droplet PCR (ddPCR) analysis revealed down-regulation of *Arx*. In humans, *ARX*, *DCLK1,* and *CDLK5* were shown to be mutated in some rare epilepsy-associated disorders. We conclude that the occurrence of seizure-like events in aged mice overexpressing *ORAI1* might be due to the down-regulation of *Arx,* and possibly of *Cdkl*5 and *Dclk1* genes.

## 1. Introduction 

Store operated calcium entry (SOCE) is a well-described process in non-excitable cells. It was initially known under the name “capacitative Ca^2+^ entry” [1] and has recently been identified also in neurons and called as nSOCE [2,3,4,5]. In the 1980s, several groups observed a phenomenon that decreasing intracellular [Ca^2+^] activates a transmembrane Ca^2+^ current [6,7,8]. During the next two decades of studies, the scientific community established molecular basis of SOCE (reviewed in [9]). Reduction in [Ca^2+^]_ER_ results in activation of plasma membrane Ca^2+^ channels that mediate sustained Ca^2+^ influx, which is required for a variety of processes. They include maintaining Ca^2+^ oscillations [10], mitochondrial fatty acid oxidation [11], refilling of Ca^2+^ stores, and a few other [12]. The essential components of SOCE are stromal interaction molecules (STIM1 and STIM2), which function as sensors of the Ca^2+^ concentration inside the endoplasmic reticulum (ER) [13,14] and ORAIs (ORAI1-3), which are highly selective Ca^2+^ channels at the plasma membrane (PM) [15,16]. Upon store depletion, STIMs form multimers, which translocate within the endoplasmic reticulum (ER) membrane towards ER-PM junctions, where they activate highly Ca^2+^-selective Ca^2+^-release-activated Ca^2+^ channels (CRACs) – ORAIs [17]. CRAC channels function as hexamer complexes that are composed of potentially multiple ORAI homologues [18,19]. Heteromeric channels that are built of endogenous ORAI1 and ORAI2 homologues were found in murine T-cells [20] and in mast cells [21], whereas in the enamel cells [22], dorsal root ganglion (DRG) neurons [23] and in overexpression studies, ORAI1:ORAI3 multimers were observed [24]. In murine immune cells, deletion of *Orai1* reduces, whereas deletion of *Orai2* increases SOCE, which indicates that ORAI2 is a less conductive subunit of CRAC channels and enables shaping of the magnitude of SOCE and modulation of the immune response [20]. Eckstein et al. postulated that ORAI1 and ORAI2 were the main channel subunits of CRAC channels in enamel cells, noting that ORAI3 can act as an inhibitor of ORAI1 in these cells [22]. In contrast, knockdown of both *Orai1* and *Orai3* in DRG neurons reduced SOCE compared with *Orai1* or *Orai3* knockdown alone [23]. These manipulations resulted in altered neuronal excitability [23]. All of these studies have shown that homo- and heteromeric CRAC channels demonstrate different SOCE response, which indicates the importance of the stoichiometry of different ORAI homologues that form the CRAC channel. The expression profile of all three homologues of ORAI varies in different tissues, with the highest level of ORAI1 in immune cells [25] and ORAI2 in the brain [26]*,* whereas ORAI3 is abundant in many solid organs and its enhanced gene expression is reported in cancerous cells [27]. Physiologically, the best-characterized homolog is ORAI1 [28,29]. Patients with inherited null or loss of function mutations in *ORAI1* suffer from severe combined immunodeficiency-like disease that is accompanied with chronic, often lethal infections and a variety of non-immunological symptoms [30,31]. 

Our recently published results indicate that overexpression of *ORAI1* in the murine brain leads to spontaneous occurrence of seizure-like events in aged animals [32]. In the present study, we extended the phenotyping of the established transgenic line overexpressing *ORAI1* in neurons (FVB/NJ-Tg(ORAI1)Ibd; Nencki Institute of Experimental Biology) by the analysis of Ca^2+^ response in the hippocampal CA1 pyramidal cells and RNA sequencing (RNAseq) gene expression profiling. Differential expression of selected genes was next confirmed by real-time PCR and validated using the digital droplet PCR (ddPCR) approach. The observed changes in Ca^2+^ signals after glutamate treatment or after ER Ca^2+^ store depletion suggest a new potential site of action for ORAI1. Our observations were supported by the identification of a set of genes, whose expression was changed in FVB/NJ-Tg(ORAI1)Ibd mice. For some of these genes, such as cyclin-dependent kinase-like 5 *Cdkl5*, aristaless related homeobox *(Arx)*, and doublecortin-like kinase 1 (*Dclk1)*, mutations in their human homologoues that lead to rare epilepsy-associated disorders were reported. Together, our results provided novel insight into the Ca^2+^ dependent actions evoked by CRAC channels that might underlie the occurrence of seizure-like episodes in FVB/NJ-Tg(ORAI1)Ibd mice.

## 2. Results

### 2.1. Overexpression of ORAI1 in Neurons Leads to Altered Ca^2+^ Response in a Modified Ca^2+^ Addback Assay in CA1 Hippocampal Region 

**Figure 1:**
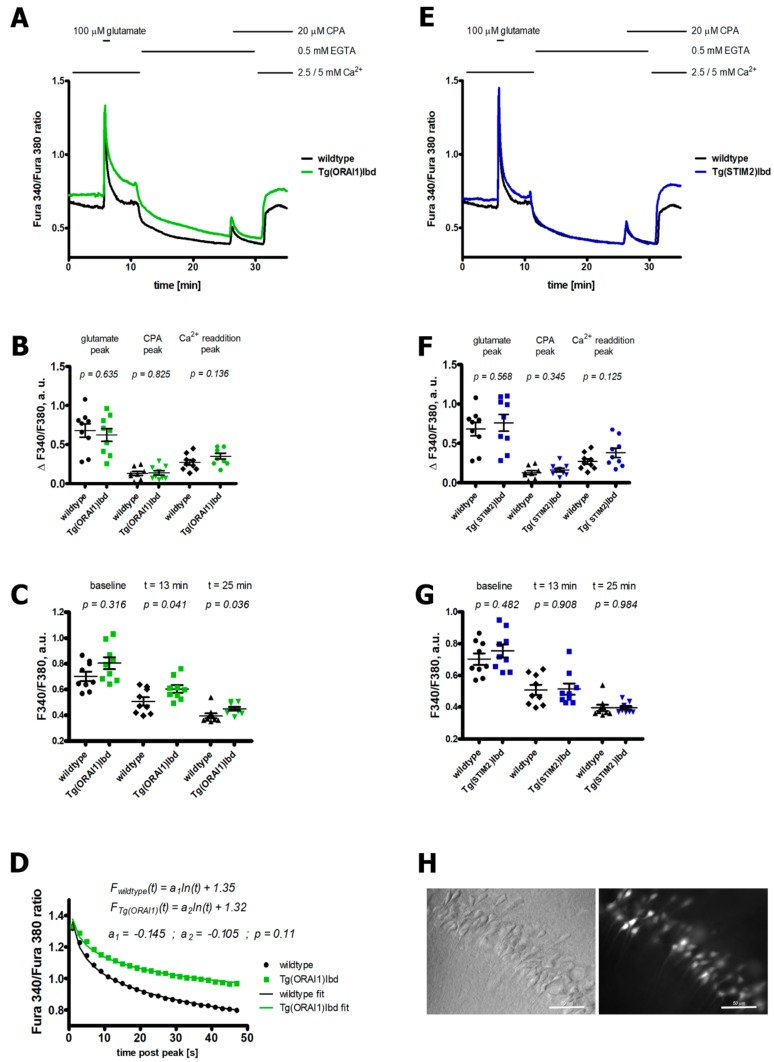
Changes in Ca^2+^ responses in the hippocampal neurons overexpressing *ORAI1* calcium channel (**A**–**D**) or stromal interaction molecule 2 (*STIM2*) (**E**–**G**) compared with wild-type neurons. Ca^2+^ measurements were performed using Fura-2 acetomethyl ester (Fura-2 AM) indicator that was loaded into the CA1 pyramidal neurons of the hippocampal acute brain slices (**H**). Typically, ~20 pyramidal neurons per one slice (n) were analyzed; the slices were isolated from at least 5 animals per genetic variant. The total number of slices analyzed per each genetic variant was 9. (**A**,**E**) Averaged time-course of background-subtracted fluorescence signal (expressed as F340/F380) from slices overexpressing *ORAI1* and *STIM2*, respectively. (**B**,**F**) Quantification of signal amplitudes observed following glutamate, cyclopiazonic acid (CPA), and Ca^2+^ (re)addition in *ORAI1* and *STIM2* overexpressing neurons, respectively, arbitrary units (a.u.). (**C**,**G**) Quantification of F340/F380 values at baseline (average of 0–5th min of the measurement) and following application of ethylene glycol-bis(β-aminoethyl ether)-*N*,*N*,*N*′,*N*′-tetraacetic acid (EGTA) (at 13th and 25th min of the measurement) in *ORAI1* and *STIM2* overexpressing neurons, respectively, a.u. (**D**) Time-course of signal decay following glutamate application in *ORAI1* and wild-type neurons that was fit by a logarithmic function. Student’s *t*-test was used to check statistical significance of the observed differences; p-values are displayed above the respective charts.

Acute brain slices of hippocampi of wild-type and transgenic *ORAI1* mice were analyzed by fluorescent imaging. Calcium measurements were performed using Fura-2 acetomethyl ester (AM) indicator that was loaded into the cells by gradual pipetting over the slice with the use of an automated perfusion system. To assess Ca^2+^ homeostasis in the *ORAI1* overexpressing neurons, we used a Ca^2+^ addback assay that was modified to enable the measurement of Ca^2+^ release from the ER [33]. According to suggestions that were proposed by Amrhein et al. (2019), the results are presented as raw *p*-values [34]. Also, the asterisks that are commonly used to signify a particular confidence interval are not shown. As shown in Figure 1A, the basal level of Ca^2+^ was slightly higher in neurons of transgenic *ORAI1* mice. Stimulation by glutamate led to comparable peaks in all genetic variants tested (Figure 1B,F). However, the time-course of signal decay following the brief pulse of glutamate tended to be slower in *ORAI1* overexpressing neurons (Figure 1D). The resulting difference in Ca^2+^ levels persisted during the subsequent application of ethylene glycol-bis(β-aminoethyl ether)-*N*,*N*,*N*′,*N*′-tetraacetic acid (EGTA) (Figure 1A,C). These changes were absent in neurons with overexpression of *STIM2* (Figure 1E,G). This may suggest that neurons of *ORAI1* transgenic mice exhibit changes in the mechanisms that are responsible for extrusion of Ca^2+^ from the cytosol by plasma membrane calcium ATP-ase (PMCA) or Ca^2+^ uptake into the ER. Moreover, a tendency towards the increased influx of Ca^2+^ ions upon Ca^2+^ addback was observed both in *ORAI1* and *STIM2* overexpressing neurons. 

### 2.2. RNAseq Analysis of Transcripts from Hippocampi Indicates Up-Regulation of the Machinery Involved in Ribosome Biogenesis and Ribonucleoprotein Complex Assembly

**Figure 2:**
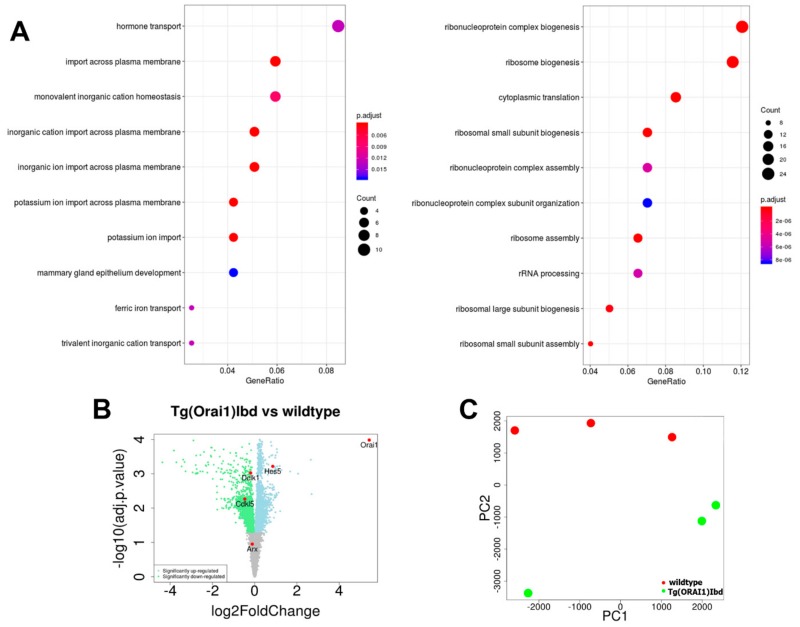
RNA sequencing (RNAseq) analysis using Noiseq tools based on data gathered from hippocampi of wild-type and FVB/NJ-Tg(ORAI1)Ibd females. (**A**) Dot plot of Gene Ontology Biological Process groups (GO BP), which are down-regulated and up-regulated in transgenic line (on the left and right, respectively). (**B**) Volcano plot represents differential gene expression between the tested variants. (**C**) Principal component analysis (PCA) of principal component 1 (PC1) and 2 (PC2). Transgenic and wild-type mice are color-coded in green and red, respectively. PCA analysis was carried out from FPKM (Fragments Per Kilobase Million) normalized RNAseq data using prcomp function in R software.

We next isolated total RNA from hippocampi of wild-type (*n* = 3, age 7 months) and of FVB/NJ-Tg(ORAI1)Ibd (*n* = 3, age 7 months) females and performed RNAseq experiments. Based on the collected RNAseq data, we performed functional (pathways, gene groups) analysis of the genes that were differentially expressed between the hippocampi of wild-type and of FVB/NJ-Tg(Orai1)Ibd females. As shown by the Gene Ontology Biological Process groups (GO BP) (Figure 2A, left), the most down-regulated genes were those belonging to hormone transport group and related with import across plasma membrane and ion transport. Most of the up-regulated genes belonged to the ribonucleprotein complex biogenesis, ribosome biogenesis, and cytoplasmic translation (Figure 2A, right). To extract the complex association between the identified GO BP groups we used the category netplot function (cnetplot). The cnetplot depicts the linkages of genes and biological concepts (GO terms) as a network, which allows to visualize the genes that are involved in the enriched pathways and genes that may belong to multiple annotation categories (Figure S1). Among the up-regulated genes, *Orai1* was present, which confirms the identity of transgenic mice (Figure 2C). The lists of genes that were identified among the particular GO BP groups and that are present in dot plots in Figure 2A are listed in data Supplementary data (Tables S1 and S2). The expression of selected genes, some of which are indicated in the volcano plot (Figure 2B), was further validated by the real-time PCR and ddPCR methods. 

### 2.3. Validation of RNAseq Data Using Customized Real-Time PCR Assays and ddPCR Technique Indicates Differential Expression of Dclk1, Cdkl5, and Arx Genes in the Hippocampi of Tg(ORAI1)Ibd Females 

We next performed real-time PCR analysis to verify the RNAseq data using customized arrays designed for the 23 genes that are listed in Table 1.

**Figure 3:**
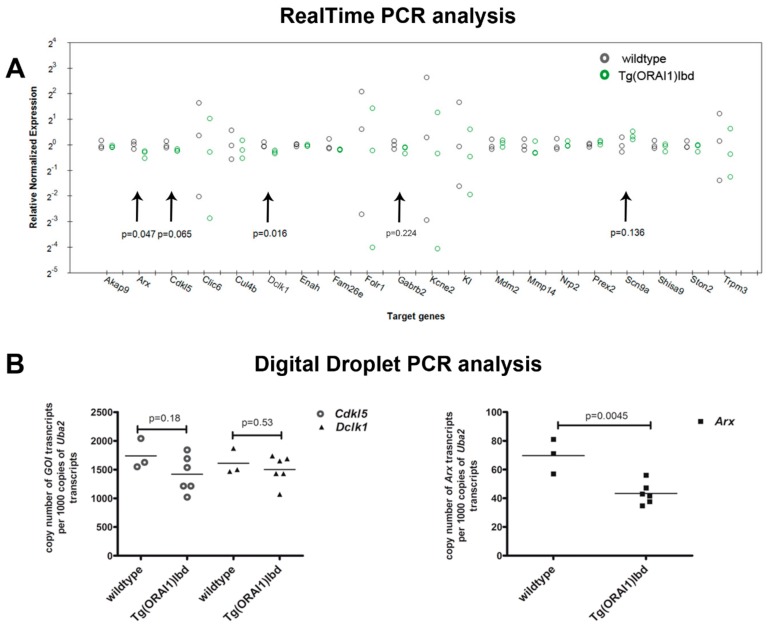
Expression profile of selected genes in hippocampi of the tested mouse variants. (**A**) Rea-time PCR analysis of the selected genes that was based on RNAseq data. The results are presented as scatter plot, each circle corresponds to one animal. Black circle corresponds to each tested wild-type probe, whereas green corresponds to transgenic ones. The obtained results suggest a down-regulation of expression of *Arx*, *Cdkl5,* and *Dclk1*. The data are presented as a fold change (2^−∆∆Ct^) normalized to the three reference genes (*Uba-2*, *Gapdh*, *Actin*). The statistics was estimated by CFX software (Bio-Rad) using the post hoc method (Tukey’s test). (**B**) The ddPCR analysis of *Arx*, *Cdkl5*, *Dclk1* using customized assays (Bio-Rad). The number of transcript copies of the selected genes are presented in relation to 1000 copies of the reference gene *Uba-2*. The results are presented as dot plot, where each sign corresponds to an individual animal (3 wild-type, 6 transgenic). The statistics were calculated by GraphPad Prism software using unpaired *t*-test. The number of *Arx* transcripts was about 30 times lower than that of *Cdkl5* and *Dclk1*, therefore, it is presented on a separate chart.

As the reference genes, *Uba-2* (catalog no. qMmuCID0018845, Bio-Rad), *Gapdh* (catalog no. qMmuCED0027497, Bio-Rad), and *Actin* (catalog no. qMmuCED0027505, Bio-Rad) were selected, which meet the criteria of the reference gene selection tool (CFX software, Bio-Rad). RNA was isolated from hippocampi of wild-type (*n* = 3, age 5 months) and of FVB/NJ-Tg(ORAI1)Ibd females (*n* = 3, age 6 months). The majority of genes showed a similar level of expression (Figure 3A and Table 1) that was calculated as 2^−∆∆Ct^. The genes which were markedly down-regulated in the transgenic individuals included *Arx* (*aristaless related homeobox*) (2^−∆∆Ct^ = 0.78, *p* = 0.047), *Dclk1* (*Serine/threonine-protein kinase*) (2^−∆∆Ct^ = 0.82, *p* = 0.016), and *Cdkl5 (Cyclin-dependent kinase-like 5)* (2^−∆∆Ct^ = 0.85, *p* = 0.02 when only *Actin* was used in the calculations as the reference gene). Human mutations in these genes are associated with epilepsy. Also *Gabrb2* (*Gamma-aminobutyric acid receptor subunit beta-2*) and *Scn9a* (*Sodium channel protein type 9 subunit alpha*) genes, which are implicated in epilepsy, showed altered expression in the transgenic animals (2^−∆∆Ct^ = 0.87, *p* = 0.23; 2^−∆∆Ct^ = 1.26, *p* = 0.136, respectively). Because the observed changes in 2^−∆∆Ct^ values were relatively modest, to validate the changes in expression of the selected genes we used a novel technique, digital droplet PCR (ddPCR, Bio-Rad). This powerful method is based on partitioning of one PCR reaction mixture into up to 20,000 independent reactions, which resulted in high precision, sensitivity, and accuracy of the data [35]. To fulfill the requirements of validation, the RNA from hippocampi was isolated from the next group of animals (3 wild-type vs. 6 transgenic, age-matched). Using customized assays that were dedicated to ddPCR analysis and *Uba-2* gene as a reference, we observed a reduction in the copy number of *Arx* (43 vs. 69, *p* = 0.0045) in transgenic females. The differences in the copy number of *Cdkl5* and *Dclk1* were insignificant (1470 vs. 1739, *p* = 0.18 and 1501 vs. 1612, *p* = 0.53, respectively). 

### 2.4. Expression of Hes-5, a Transcriptional Repressor That Is Involved in Neurogenesis, Is Up-Regulated in Tg(ORAI1) Ibd Females 

In the next set of the real-time PCR analysis based on RNAseq data, we designed an additional 15 pairs of primers (Table 2).

**Figure 4:**
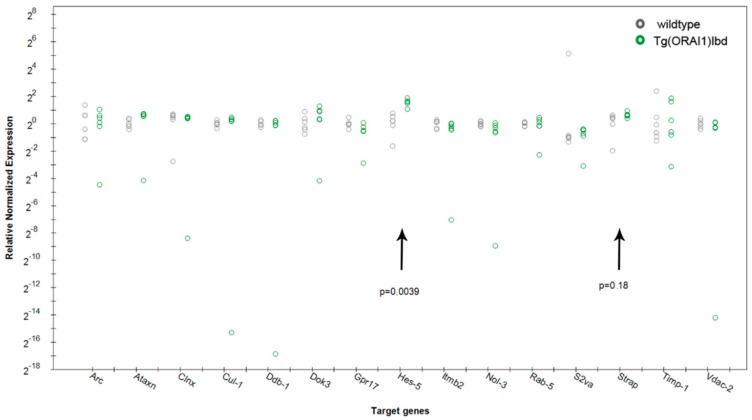
Expression profile of the selected genes in the hippocampi of the tested mouse variants that was obtained by real-time PCR analysis. The results are presented as scatter plot; each circle corresponds to one animal. The obtained results suggest an up-regulation of the expression of *Hes-5* and *Strap*. The data are presented as a fold change (2^−∆∆Ct^) that was normalized to the reference gene (*Uba-2*), the statistics was estimated by CFX software (Bio-Rad) using post hoc method (Tukey’s test).

Using the matrixes that were derived from previous experiments (6 wild-type vs. 6 transgenic mice) and *Uba-2* as a reference gene*,* we identified an up-regulation of *Hes-5* (Transcription factor *HES-5*) expression (2^−∆∆Ct^ = 2,93, *p* = 0.0039) (Figure 4). Among the genes that were analyzed and described in Table 2, we detected a trend towards the up-regulation of *Strap* (Serine-threonine kinase receptor-associated protein). The protein is a part of the survival of motor neurons (SMN) complex that acts as a catalyst in the assembly of small nuclear ribonucleoproteins (snRNPs), the building blocks of the spliceosome (2^−∆∆Ct^ = 1.56, *p* = 0.18).

## 3. Discussion

The role of SOCE in neurons and its components, such as STIM proteins and Ca^2+^ channels, is far from being understood. It becomes clear that not only ORAI and transient receptor potential cation channels (TRPCs) [36], but also voltage-gated dependent channels (VGCC) [37] and α-amino-3-hydroxy-5-methyl-4-isoxazolepropionic acid (AMPA) receptors [38,39] are sensitive to STIMs. Our group also demonstrated the involvement of STIM1 in mGlur-LTD phenomenon in CA3-CA1 projection of hippocampus in the novel FVB/NJ-Tg(STIM1)Ibd transgenic line [40]. On the other hand, it is now well established that local Ca^2+^ microdomains that appear upon opening of store operated calcium channels activate downstream effectors like calcineurin or adenylyl cyclase in non-excitable cells. The wide scope of processes mediated by Ca^2+^ nanodomains that are generated by SOCE is well described in a recent review by Barak and Parekh [9]. 

In hippocampal neurons nSOCE is believed to be mediated mainly by STIM2 together with Orai2 and Trpc6. It was suggested that Ca^2+^ that is delivered by nSOCE is responsible for Ca^2+^/calmodulin-dependent protein kinase II (CaMKII) pathway activation, which contributes to spine maintenance of mushroom spines [41]. Ca^2+^ disruption leads to synaptic loss and cognitive decline in aging and Alzheimer’s disease [41,42,43]. The role of nSOCE seems to be even more complex as indicated by the recent results by Chen–Engerer et al. [26]. They demonstrated that in CA1 pyramidal cells nSOCE that is mediated by ORAI2 is essential for refilling of IP3 sensitive Ca^2+^ stores, whereas ryanodine-sensitive stores are VGCC dependent. The authors postulate that their observations may help to explain the distinct roles of inositol triphosphate receptor (IP3Rs) and RyRs for various forms of synaptic plasticity. 

As a consequence of *ORAI1* transgene overexpression in mice used in our studies, we modified the level of ORAI1 in neurons, especially in the hippocampus, in which the predominant isoform is ORAI2. The observed trend of a higher amplitude of nSOCE in neurons of Tg(ORAI1)Ibd transgenic mice is in line with the observation by Vaeth et al. (2017), who showed that ORAI2 was a less conductive subunit of CRAC channel in immune cells [20]. This observation was supported by Fura- 2 quenching experiments using Mn^2+^, in which we observed faster decay of the signal in *ORAI1* overexpressing neurons (Figure S2). Because Mn^2+^ quenches the fluorescence of Fura-2 and store-operated channels are permeable to them, such approach can be used to assess the activity of SOCs. Moreover, a trend for different kinetics of the [Ca^2+^] decay after glutamate treatment and the subsequent significant differences in the Ca^2+^ level in chelating conditions that were described in this work are in agreement with the observations done by Chen–Engerer et al. (2019). They showed the importance of ORAI2-mediated nSOCE in refilling of IP3 sensitive Ca^2+^ stores [26]. Due to the fact that glutamate stimulation also evokes IP3 signaling cascade that in turn leads to Ca^2+^ release from the ER, subsequent ORAI-dependent refilling of ER Ca^2+^ stores might be altered in neurons of our transgenic line [44]. These observations suggest that our transgenic line might be a useful tool for an in-depth analysis of specific molecular targets that are activated by nSOCE in the brain. 

RNAseq analysis of the material from Tg(ORAI1)Ibd transgenic line identified up-regulation of the genes that belong to the GO biological processes that are involved in the ribonucleoprotein complex biogenesis, ribosome biogenesis, and assembly, which are all components of translational machinery. It was demonstrated in acinar polarized cells that overexpression of STIM1 increased the density of ribosome-free terminals in rough ER (RER), and facilitated the formation of ER-plasma membrane (ER-PM) junctions and SOCE propagation. If our future validation of these RNAseq data by real-time and ddPCR is positive, it may suggest that Ca^2+^ microdomains that are generated by nSOCE serve as a regulatory component of the translation process [45,46]. 

As previously reported by our group, overexpression of ORAI1 in neurons induced seizure-like symptoms in aged female mice [32]. Application of kainic acid and bicuculline to hippocampal slices from Tg(ORAI1)Ibd females revealed a significantly lower frequency of interictal bursts compared with samples that were isolated from wild-type female or transgenic male mice. The down-regulation of *Arx*, *Cdkl5,* and *Dclk1* genes that we observed in the present work is in line with the report that loss of function mutations of human aristaless related homeobox (*ARX*)*, CDKL5,* and *DCLK1* are found in epileptic patients [47] (Table 3). It might be speculated that the Ca^2+^ nanodomains that appear locally by the activity of CRAC channels affect the expression of these genes via downstream pathways.

In conclusion, our data indicate that overexpression of ORAI1 changed the subunit composition of CRAC channels, which caused differences in the observed Ca^2+^ responses and induced specific changes in gene expression. By real-time PCR, we detected a down-regulation of *Arx*, *Cdkl5,* and *Dclk1* genes, human homologues of which are involved in etiology of epilepsy. This down-regulation might explain seizure-like phenomena that were observed in 15-month-old females of the FVB/NJ-Tg(ORAI1)Ibd line. 

## 4. Materials and Methods

### 4.1. Animal Care

The mice were housed under standard conditions on a 12 h/12 h light/dark cycle with food and water available ad libitum. All of the animal experiments were approved by the Local Commission for the Ethics of Animal Experimentation No. 1 in Warsaw (approval no. 756/2015, 25 May 2015 and approval no. 416/2017, 21 November 2017) and performed in accordance with the European Communities Council Directive (63/2010/EEC, 22 September 2010). All analyses described in the text were performed on 7 ±1-months-old female mice. 

### 4.2. Generation of FVB/NJ–Tg(ORAI1)Ibd Transgenic Mice and Characterization of Transgene Expression

The details on the generation of the transgenic lines were described elsewhere [32]. In brief, a 1-kb coding sequence of human ORAI1 (cDNA clone MGC:21530 IMAGE:3914595; Dharmacon, USA) was amplified by PCR with XhoI-containing primers (forward 5′-ACTTCACTCGAGACCATGCATCCGGAGCCCG-3′, reverse 5′- ACTTCACTCGAGCTAGGCATAGTGGCTGCCG-3′). The PCR product was digested with *XhoI* and subcloned into the *Xho*I site of the modified mouse Thy-1.2 expression cassette [66,67]. The Thy1.2-ORAI1 construct was subsequently digested with *PvuI* and *NotI* and used for typical pronuclear microinjection [68]. Offspring of the F2 generation and onward were genotyped by PCR using the following primers: Thy_Forw_genotype 5′-TCTGAGTGGCAAAGGACCTTAGG-3′ and ORAI1_Rev_genotype 5′-TGGTCCTGTAAGCGGGCAAAC-3′.

### 4.3. Brain Slice Preparation

The reagents were purchased from Sigma unless indicated otherwise. Hippocampal sections were prepared according to the protective recovery method [69]. Mice of the age P25 ± 5 were sacrificed by cervical dislocation. Next, brains were isolated and placed in a dish filled with N-methyl-D-glucamine (NMDG)-based artificial cerebrospinal fluid (NMDG-aCSF) solution that was cooled to ~2°C and contained, in mM: NMDG, 92, KCl, 2.5, 4-(2-hydroxyethyl)-1-piperazineethanesulfonic acid (HEPES), 20, thiourea, 2, glucose, 25, NaH_2_PO_4_, 1.25, NaHCO_3_, 30, MgSO_4_, 10, CaCl_2_, 0.5, Na-ascorbate, 5, Na-pyruvate, 3 N-acetyl-L-cystein (NAC, myprotein.com, Manchester, United Kingdom), 3. Sections that were 350 μm thick were obtained with the use of a vibratome (HM 650V, Thermo Scientific, Waltham, MA, USA). During the slicing procedure, the tissue was submerged in ice-cold NMDG-aCSF solution that was bubbled with carbogen (95%/5% O_2_/CO_2_). Next, the sections were incubated in carbogenated NMDG aCSF for 10 min (temperature: ~33 °C). A 2M NaCl solution was gradually added to the solution according to [69]. The slices were then transferred to HEPES-based aCSF that contained, in mM: NaCl, 82, KCl, 2.5, HEPES, 20, thiourea, 2, glucose, 25, NaH_2_PO_4_, 1.25, NaHCO_3_, 30, MgSO_4_, 2, Na-ascorbate, 5, Na-pyruvate, 3, and 12 mM NAC (temperature: ~25 °C). The recordings were started after 1 h of incubation in HEPES-aCSF. The pH of all of the solutions was between 7.3 and 7.4. 

### 4.4. Dye-Loading Procedure

A 1 mM stock of Fura-2 AM (Thermo-Fisher) that was dissolved in DMSO was diluted in artificial cerebrospinal fluid (aCSF) (it contained, in mM: NaCl, 126, KCl, 2.2, glucose, 20, NaH_2_PO_4_, 1.25, NaHCO_3_, 25, MgSO_4_, 1, and CaCl_2_, 2.5; the pH was adjusted to 7.3–7.4) to a final concentration of 50 µM. The solution was supplemented with Pluronic F-127 (Sigma-Aldrich, St. Louis, MO, USA) at a final concentration of 2.5%. The dye solution was applied locally above the hippocampal slice with the use of Perfusion Pencil (Automate Scientific, Berkeley, CA, USA), for 20 min. During the loading procedure, the slices were perfused with aCSF solution at a rate of 4–5 mL/min, at 33 °C, to facilitate incorporation of the dye molecules by the cells. After the loading, the slices were allowed to rest for 20 min, under a constant perfusion of aCSF at a temperature of 25 °C. The consistency of the loading procedure was checked by acquiring fluorescent Fura-2 signal at its isosbestic point (λ = 360 nm). The intensity of the signal excited at this wavelength should not depend on Ca^2+^ concentration and thus only reflect the quantity of the incorporated and de-esterified dye alone. The signal was comparable across preparations. Moreover, we did not detect any correlation between the loading efficiency and the magnitude of the recorded Ca^2+^ responses.

### 4.5. Ca^2+^ Imaging of Neurons in Brain Slices

The measurements were performed using MetaFluor software, a Zeiss Examiner Z.1 microscope (40×/1.0 objective), and the Retiga ELECTRO™ CCD Camera (Teledyne Qimaging, Surrey, BC, Canada). A peristaltic pump was used to perfuse the hippocampal slices with aCSF that contained 126 mM NaCl, 2.6 mM KCl, 20 mM glucose, 1.25 NaH_2_PO_4_, 25 mM NaHCO_3,_ and 1 mM MgSO_4_. Additionally, aCSF that contained chemicals used for evoking Ca^2+^ responses was applied locally through a 100 µm wide tip that was positioned 100 µm above the pyramidal neurons of the CA1 hippocampal area. The pencil was connected to an automated perfusion system ValveLink 8.2 (Automate Scientific, Berkeley, CA, USA). For the first 10 min of the recordings (baseline recording, glutamate application, and signal relaxation), 2.5 mM CaCl_2_ was included in the solution that was perfused with the use of the pump. Next, the solution was manually changed to Ca^2+^-free aCSF that contained 0.5 mM (ethylene glycol-bis(β-aminoethyl ether)-*N*,*N*,*N*′,*N*′-tetraacetic acid) EGTA. The pH of aCSF was adjusted to 7.4 at room temperature with carbogen and HCl. The extracellular solution was carbogenated throughout the recordings and heated with an in-line heater (catalog no. 64-0102, controlled by a TC-324B temperature control unit, Warner Instruments) to maintain the temperature in the recording chamber close to 25 °C. The rate of solution flow was 4–5 mL/min.

To assess Ca^2+^ homeostasis in the CA1 pyramidal neurons of the hippocampus, a Ca^2+^ addback assay that was modified to allow for the quantification of the Ca^2+^ efflux from the ER [33] was used. Changes in the fluorescent signal in the neuronal cell bodies were monitored and quantified. For baseline recording, aCSF that contained 2.5 mM Ca^2+^ was perfused for 5 min, which was followed by the local application of 100 µM Na-glutamate for 10s. Next, the signal relaxation was monitored for another 5 min in 2.5 mM Ca^2+^ aCSF. To deplete the extracellular milieu from the Ca^2+^ ions, the solution was changed to aCSF that contained 0.5 mM EGTA for 15 min, which was followed by the local application of 20 μM cyclopiazonic acid (CPA) for 5 min. Next, Ca^2+^ was re-added to the solution at a concentration of 5 mM Ca^2+^ for 5 min, in the presence of 20 μM CPA. To limit activity-driven Ca^2+^ entry, 1 μM Tetrodotoxin (TTX) and 10 μM nifedipine were included in all solutions that were used from the 10th min of the experiment onwards. 

For Fura-2 quenching experiments, hippocampal slices were loaded with the dye as described above. For baseline recording, Ringer’s solution (composed of, in mM: NaCl, 137, KCl, 5, glucose, 24, HEPES, 10, CaCl_2_, 2, MgCl_2_, 1, pH = 7.3–7.4) was perfused for 5 min, which was followed by application of Ca^2+^-free Ca^2+^ depleting solution (Ringer’s solution supplemented with 0.5 mM EGTA and 20 μM CPA) for 15 min. Next, Ca^2+^-free Ringer’s solution supplemented with 2 mM Mn^2+^ (as MnCl_2_) was locally applied above the cells of interest, in the absence of EGTA and presence of 20 μM CPA. To limit activity-driven Ca^2+^ entry, 1 μM TTX and 10 μM nifedipine were included in all solutions. To quantify the rate of Mn^2+^ influx, linear part of the curves following Mn^2+^ addition (which corresponded to the first 25 s of treatment) were approximated with a linear function and the decay slope was calculated (all calculations performed in Microsoft Excel).

### 4.6. Data Analysis 

The acquired signals were quantified and exported with the use of MetaFluor software and further analyzed with Microsoft Excel and GraphPad Prism 5 (USA). Student’s *t*-test was used to check the statistical significance of the observed differences between the groups.

### 4.7. Global Gene Expression Profiling

Total RNA was extracted from three individual hippocampi per variant using RNeasy Lipid Mini Kit (Qiagen: 74804, Hilden, Germany) and treated on-column with DNase using the RNase-Free DNase Set (Qiagen: 79254). Preparation of cDNA libraries and sequencing by Illumina HighSeq 1500 (run type: Paired-end sequencing, read length: 1 × 76 bp) were carried out in cooperation with Core Facility of IIMCB and the Nencki Institute. Sequencing reads (fastq) were filtered by Trimmomatic program, discarding all reads contaminated by sequencing adapter sequence as well as discarding reads whose mean quality in a bin of 20 base pair (bp) was below Q30. Minimal length of read used for mapping was set as 65 bp. Quality filtered reads were aligned to mouse genome (mm10) by STAR program. Statistical analyses, including comparisons to other samples, were done in R environment using NOIseq library [70]. Functional analysis was performed for the genes that were differentially expressed in transgenic versus wild-type samples (raw *p*-value below 0.05), separately for genes up- and down-regulated in transgenic animals. The functional analysis of data was performed using clusterProfiler [71] to find statistically significant Gene Ontology terms. The whole metadata of RNAseq analysis deposit in NCBI’s Gene Expression Omnibus [72] and are accessible through GEO Series accession number GSE138370 (https://www.ncbi.nlm.nih.gov/geo/query/acc.cgi?acc=GSE138370).

### 4.8. Real-Time Polymerase Chain Reaction Arrays and Quantitative Real-Time PCR (qPCR)

Hippocampi were dissected from three adult female mice (≥ postnatal day 180 (PD180)) per variant for real-time polymerase chain reaction arrays. The material was homogenized in Qiazol (Qiagen, catalog no. 79306, Germany) and subjected to total RNA isolation according to manufacturer’s protocol of RNeasy Lipid Tissue Mini Kit (Qiagen: 74804, Germany). The quality of RNA was checked by absorbance measurements at 260, 280, and 230 nm. For the analysis, only samples whose A260/280 nm and A230/280 nm values exceeded 1.8 were used. There were 1000 ng of RNA templates used to synthesize first-strand cDNA, with the use of iScript reverse transcription supermix (Bio-Rad: 170–8841, USA). The 96-well custom plates that contained primers for target and reference genes (Bio-Rad, USA) and SsoAdvanced Universal SYBR Green Supermix (Bio-Rad:1725274; USA) were used to perform teal-time polymerase chain reaction (RT-PCR, carried out in duplicate). The cDNA (50 ng) was used for each reaction. Small Ubiquitin-like Modifier (SUMO)-activating enzyme subunit 2 (*Uba-2)*, glyceraldehyde-3-phosphate dehydrogenase (*Gapdh*), and *Actin (Actb)* were used as reference genes. The reactions were performed using the CFX Connect™ Real-Time PCR Detection System (Bio-Rad). Bio-Rad CFX Maestro 1.1 software was used to analyze the data (Bio-Rad, USA). The reference genes’ stability was acceptable (as evaluated by the reference gene selection tool). For calculation of the expression levels, equal efficiencies were assumed for all reactions and the 2^−∆∆Ct^ method was used. For each gene, the average normalized expression was calculated from the following formula:

$$\mathrm{Normalized}\;\mathrm{expressionGOI}\;=RQ\mathrm{GOI}/{\left(RQUba2\times RQGapdh\times RQActb\right)}^{\frac{1}{3}}$$

where: GOI, gene of interest; RQ, relative quantity, calculated as RQ = 2^(−CtGOI)^.

The statistical significance of the observed differences in the expression levels between the tested groups was determined with the use of analysis of variance (ANOVA), followed by Tukey’s Honestly Significant Difference (HSD) post-hoc test.

The real-time PCR reactions were performed in duplicate for each sample using SsoAdvance Universal SYBR Green Supermix (Bio-Rad) with the following sets of specific gene primers (the primer sequences are listed in Table 2, Sigma). Based on the real-time PCR arrays, *SUMO-activating enzyme subunit 2* (*Uba-2)* was chosen as reference gene. The specificity of the reactions was determined based on dissociation curve analysis. Relative gene expression was calculated using the 2^−ΔΔCt^ method and Bio-Rad CFX Maestro 1.1 software (Bio-Rad, USA). Six adult female mice (≥ postnatal day 180 (PD180), per variant were taken to perform this experiment.

### 4.9. DdPCR Validation of Selected Genes

Primers for *Arx*, *Cdkl5*, *Dclk1*, and *Uba2* were sourced from the EvaGreen ddPCR™ assays (Bio-Rad: dMmuEG5062557; dMmuEG5078166; dMmuEG5084027; dMmuEG5081512, respectively) and diluted according to the kit instructions. For ddPCR technology (QX200 Droplet Digital PCR ddPCR™) System—Bio-Rad, USA) 21 μL reaction mixtures containing 25 ng of cDNA (six transgenics mice vs. 3 wild-type mice variant), primers, and QX200™ddPCR™ EvaGreen Supermix (Bio-Rad: 186–4034) were used. Droplet generation and transfer of emulsified samples to PCR plates (Bio-Rad: 12001925) were performed according to the manufacturer’s instructions (Instruction Manual, QX200™ Droplet Generator—Bio-Rad). The ddPCR plate was sealed with a foil heat seal (Bio-Rad, 181–4040) and the PX1™ PCR Plate Sealer (Bio-Rad; 181–4000). The following cycling protocol was established according to the manufacturer’s protocol: 95 °C enzyme activation step for 5 min followed by 40 cycles of a two-step cycling protocol (95 °C for 30 s and 58 °C for 1 min). The ramp rate between these steps was slowed to 2 °C/second. The post-cycling protocol was in accordance with the kit instructions (Bio-Rad, 186–4034). The absolute quantity of DNA per sample (copies/μL) was processed using QuantaSoft version 1.7.4.0917 (Bio-Rad, USA). The data were next exported to Microsoft Excel for further analysis. 

## Figures and Tables

**Table 1:** A list of selected genes that were chosen for validation using customized arrays. *Arx, Dclk1*, *Cdkl5* and were subsequently taken to ddPCR analysis (framed in green).

				Bio-Rad Assays	qPCR	
	Gene Name	ENSEMBL ID	Description	Cat. No.	2^−∆∆Ct^	*p*-Value Tukey
Genes associated with epilepsy	*Kcne2*	ENSMUSG00000039672	potassium voltage-gated channel, Isk-related subfamily, gene 2	qMmuCID0011027	0.487	0.670
	*Arx*	ENSMUSG00000035277	aristaless related homeobox	qMmuCED0004831	0.787	0.046
	*Dclk1*	ENSMUSG00000027797	serine/threonine-protein kinase DCLK1	qMmuCID0022222	0.827	0.015
	*Cdkl5*	ENSMUSG00000031292	cyclin-dependent kinase-like 5	qMmuCID0013381	0.868	0.064
	*Gabrb2*	ENSMUSG00000007653	gamma-aminobutyric acid (GABA) A receptor, subunit beta 2	qMmuCID0005334	0.886	0.224
	*Scn9a*	ENSMUSG00000075316	sodium channel protein type 9 subunit alpha	qMmuCID0015354	1.283	0.131
	*Folr1*	ENSMUSG00000001827	folate receptor 1	qMmuCID0016330	0.5262	0.688
	*Clic6*	ENSMUSG00000022949	chloride intracellular channel 6	qMmuCID0011099	0.615	0.678
	*Kl*	ENSMUSG00000058488	klotho, the Klotho peptide generated by cleavage of the membrane-bound isoform may be an anti-aging circulating hormone	qMmuCID0007154	0.663	0.648
	*Trpm3*	ENSMUSG00000052387	transient receptor potential cation channel, subfamily M, member 3	qMmuCID0040163	0.8	0.747
	*Fam26e*	ENSMUSG00000049872	calcium homeostasis modulator protein 5	qMmuCID0008059	0.882	0.213
	*Cul4b*	ENSMUSG00000031095	cullin 4B, Core component of multiple cullin-RING-based E3 ubiquitin-protein ligase complexes	qMmuCID0011478	0.884	0.667
	*Mmp14*	ENSMUSG00000000957	matrix metallopeptidase 14 (membrane-inserted)	qMmuCID0006120	0.889	0.473
	*Ston2*	ENSMUSG00000020961	stonin 2, adapter protein involved in endocytic machinery	qMmuCID0014604	0.936	0.462
	*Shisa9*	ENSMUSG00000022494	regulator of short-term neuronal synaptic plasticity in the dentate gyrus	qMmuCID0022663	0.940	0.523
	*Akap9*	ENSMUSG00000040407	A-kinase anchor protein 9, scaffolding protein	qMmuCID0023460	0.958	0.542
	*Enah*	ENSMUSG00000022995	ENAH actin regulator; actin-associated proteins involved in a range of processes dependent on cytoskeleton remodeling and cell polarity such as axon guidance	qMmuCID0026581	0.985	0.593
	*Nrp2*	ENSMUSG00000025969	Neuropilin-2, High affinity receptor for semaphorins	qMmuCID0005743	1.021	0.835
	*Mdm2*	ENSMUSG00000020184	E3 ubiquitin-protein ligase	qMmuCID0025320	1.044	0.676
	*Prex2*	ENSMUSG00000048960	phosphatidylinositol-3,4,5-trisphosphate-dependent Rac exchange factor 2	qMmuCID0039899	1.070	0.187

**Table 2:** List of selected genes together with primers sequence whose expression profile was analyzed using real-time PCR. Up-regulation of hairy and enhancer of split-5 (*Hes-5)*, a transcriptional repressor, was detected in the hippocampus of Tg(ORA1)Ibd line (highlighted in green).

			Primer sequence		qPCR	
Gene Name	ENSEMBL ID	Description	Forward	Revers	2^−∆∆Ct^	*p*-Value Tukey
*Ddb1*	ENSMUSG00000024740	damage specific DNA binding protein 1	TCAAAGAGGTGGGAATGTACG	GATGTCAATGCTTTCGCCAC	0.147	0.351
*Vdac2*	ENSMUSG00000021771	voltage-dependent anion channel 2	TGGAACACCGATAACACTCTG	CACTCCCTCTTGTAAGCAGAC	0.18	0.317
*Cul1*	ENSMUSG00000029686	cullin 1	ACCGATTCTCCAGCAAAGTG	TCTCCATGTCACCAATGCAAG	0.204	0.400
*Nol3*	ENSMUSG00000014776	nucleolar protein 3 (apoptosis repressor with CARD domain)	TCCAAGAAGAGGATGAATCTGAAG	ATTTGGCAGTAGGTGTCTCG	0.298	0.255
*Itmbl2*	ENSMUSG00000022108	integral membrane protein 2B	TTTGAGGAAGACGCAGTGG	TGTTCAGAGGAATCACGTAG	0.396	0.272
*Clnx*	ENSMUSG00000020368	calnexin	CTTTGCCAGTGTTCCTTGTG	CTTCCTCTTCATCCCTCTTGTTC	0.495	0.534
*Sv2a*	ENSMUSG00000038486	synaptic vesicle glycoprotein 2 a	GTGGACACTCTACTTCGTGC	ATGCCCAGGTACACAATGAG	0.502	0.395
*Gpr17*	ENSMUSG00000052229	G protein-coupled receptor 17	TCTCCTGTCCTTTCCTTCCT	TCTCTTGTCCGCATTGCTC	0.587	0.117
*Arc*	ENSMUSG00000022602	activity regulated cytoskeletal-associated protein	CTACAGAGCCAGGAGAATGAC	GTGTCTTGGAACCCATGTAGG	0.752	0.668
*Rab5*	ENSMUSG00000000711	Rab5B. member RAS oncogene family	AGGGAACAAAGCTGACCTTG	TGCCAGGAAGAGATCATTCAC	0.820	0.505
*Atxn*	ENSMUSG00000074748	ataxin 7-like 3B	TGTGTACCCAGCCTATACAATTC	CCTGACCATCAACACCATCTAA	0.906	0.865
*TIMP1*	ENSMUSG00000001131	tissue inhibitor of metalloproteinase 1	CTCAAAGACCTATAGTGCTGGC	CAAAGTGACGGCTCTGGTAG	0.913	0.890
*Dok3*	ENSMUSG00000035711	docking protein 3	ACTGGTGCCTTCCTGATTAC	GATCCTGACGAACATTCTCCG	0.961	0.949
*Strap*	ENSMUSG00000030224	serine/threonine kinase receptor associated protein	CCAGGGAGATACAGGAGACT	AGACCGCATCCCATACTTTG	1.568	0.186
*Hes-5*	ENSMUSG00000048001	hes family bHLH transcription factor 5	CTACCTGAAACACAGCAAAGC	AGCTTCATCTGCGTGTCG	2.932	0.003

**Table 3:** Phenotypes of human mutations of genes which are associated with epilepsy.

Gene Name	Encoded Protein Function	Phenotype in Human	Reference
*Arx*	aristaless related homeobox gene- regulator of gene transcription important forbrain development	Early infantile epileptic encephalopathy (EIEE); X-linked lissencephaly with abnormal genitalia (XLAG)	[48,49,50,51,52]
*Cdkl5*	X-linked serine/threonine kinase cyclin-dependent kinase-like 5	Early infantile epileptic encephalopathy (EIEE)	[53,54,55,56,57]
*Dclk1*	doublecortin-like kinase 1	Focal seizures	[58]
*Gabrb2*	subunit of γ-aminobutyric acid type A (GABAA) receptor	Generalized epilepsy with febrile seizures plus (GEFS+); Childhood absence epilepsy (CAE); Lennox-Gastaut syndrome	[59,60,61]
*Scn9a*	sodium channel	Dravet syndrome (DS); Familial febrile seizures (FFS); Generalized epilepsy with febrile seizures plus (GEFS+)	[62,63,64]
*Kcne*	a family of single-helix transmembrane proteins with 5 known members that modulate the function of several ion channels	Familial neonatal seizures	[65]

## Supplementary Materials

The following are available online at https://www.mdpi.com/1422-0067/20/22/5539/s1.

## Abbreviations

a.u.	arbitrary units
aCSF	artificial cerebrospinal fluid
AM	acetomethyl ester
AMPA	α-amino-3-hydroxy-5-methyl-4-isoxazolepropionic acid
Arx	aristaless related homeobox
CaMKII	Ca^2+^/calmodulin-dependent protein kinase II
Cdkl5	cyclin-dependent kinase-like 5, also known as serine/threonine kinase 9 (STK9)
CPA	cyclopiazonic acid
CRAC	calcium release activated channel
EGTA	ethylene glycol-bis(β-aminoethyl ether)-*N*,*N*,*N*′,*N*′-tetraacetic acid
Dclk1	doublecortin like kinase 1
ddPCR	digital droplet PCR
DRG	dorsal root ganglion
ER	endoplasmic reticulum
FPKM	Fragments Per Kilobase Million
Fura-2 AM	Fura-2 acetomethyl ester
GO BP	gene ontology biological process
Hes-5	hairy and enhancer of split-5
Ibd	Institute of Experimental Biology, in Polish: Instytut Biologii Doświadczalnej
IP3R	inositol triphosphate receptor
NMDG	N-methyl-D-glucamine
nSOCE	neuronal store operated calcium entry
PMCA	plasma membrane calcium ATP-ase
RER	rough endoplasmic reticulum
RNASeq	RNA sequencing
SOCE	store operated calcium entry
STIM	stromal interaction molecule
Tg	transgenic
TRPCs	transient receptor potential cation channels
TTX	tetrodotoxin
VGCC	voltage-gated calcium channel
