# Supplementary material for: Changes in Calcium Homeostasis and Gene Expression Implicated in Epilepsy in Hippocampi of Mice Overexpressing ORAI1
Source: Int J Mol Sci. 2019 Nov 6;20(22):5539. doi: 10.3390/ijms20225539 (PMC6888010; doi:10.3390/ijms20225539)
Supplement: Supplementary file 1 [file ijms-20-05539-s001.zip › supplementary/ijms-618780 Supplementary data.docx]

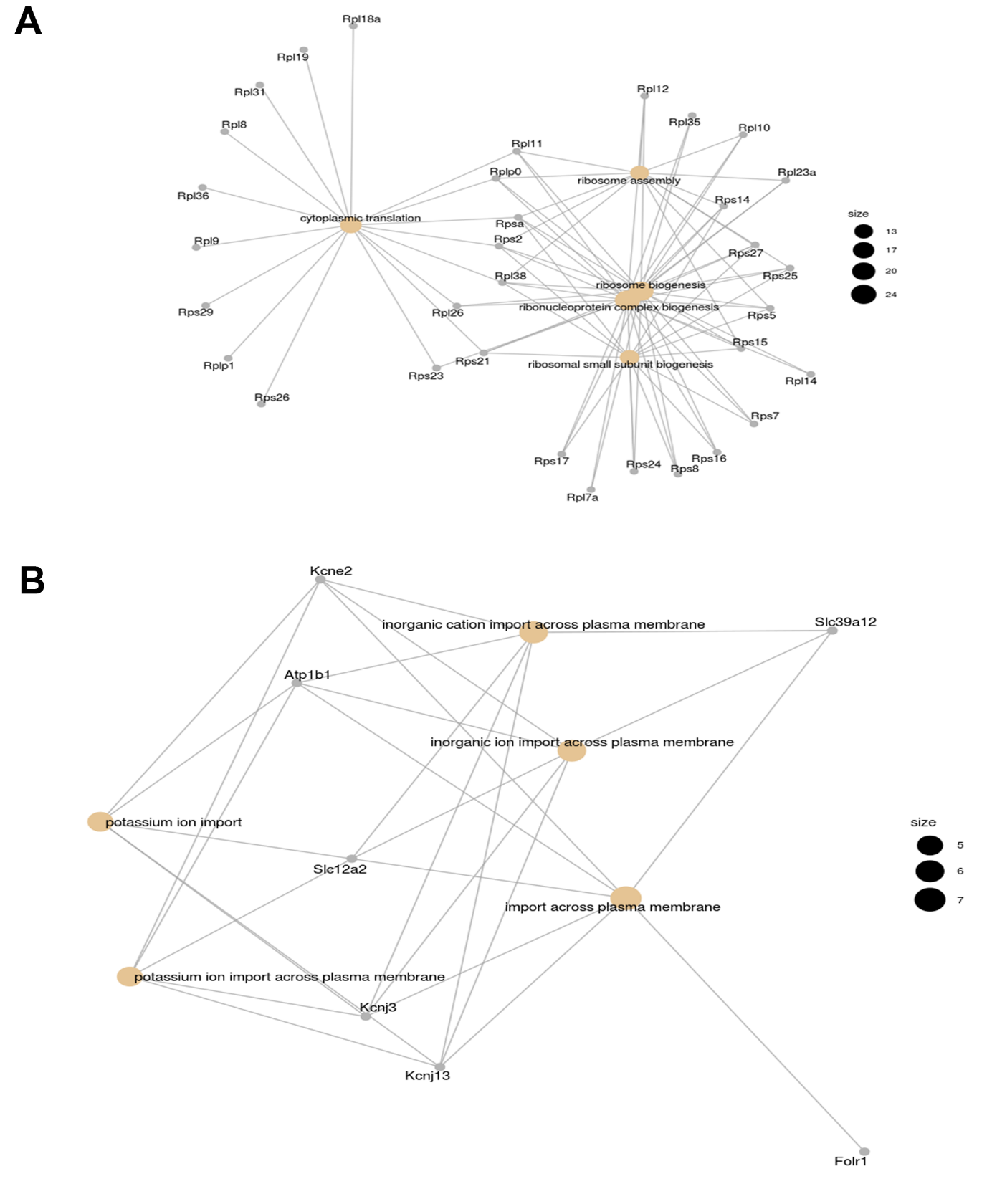


**Figure S1.** Cnetplot, plot showing a network of upregulated (**A**) and downregulated (**B**) GO BP (Biological Process) gene groups and their interconnections. Size of a dot of GO BP term corresponds to the number of genes involved. The cnetplot depicts the linkages of genes and biological concepts (GO terms) as a network, which allows to visualize the genes that are involved in the enriched pathways and genes that may belong to multiple annotation categories.


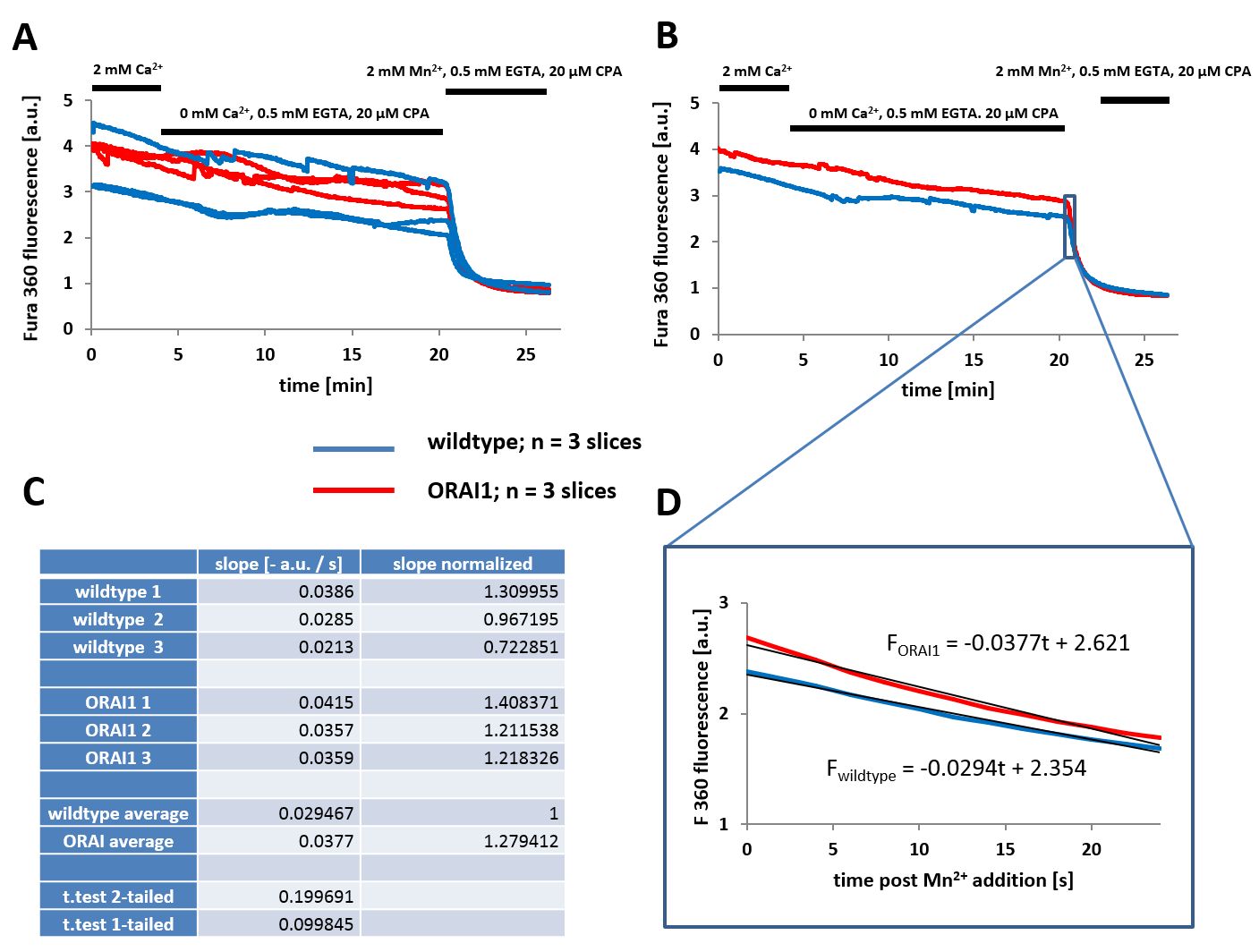


**Figure S2.** Fura-2 quenching experiment by Mn^2+^. As an alternative approach for the assessment of the activity of SOC channels we performed Fura-2 quenching experiments using Mn^2+^. A sharp decrease in the signal upon Mn^2+^ addition was observed: (**A**) – traces from single slices, each trace is an average of responses obtained from 30-40 cells; 3 replicates per variant; (**B**) – averaged traces from panel (**A**). The slopes of the linear part of the curves following Mn^2+^ addition (which correspond to the first 25 s of treatment) were compared (**D**). The results revealed a tendency towards increased decay rate in neurons overexpressing ORAI1 (quantification in **C**).
